# Using pharmacists and drugstore workers as sexual healthcare givers: a qualitative study of men who have sex with men in Dar es Salaam, Tanzania

**DOI:** 10.1080/16549716.2017.1389181

**Published:** 2017-10-26

**Authors:** Charlotte Agardh, Felicia Weije, Anette Agardh, Joyce Nyoni, Michael W. Ross, John Kashiha, Markus Larsson

**Affiliations:** ^a^ School of Medicine, Faculty of Medicine, Lund University, Malmö, Sweden; ^b^ Division for Social Medicine and Global Health, Department of Clinical Sciences, Malmö, Lund University, Malmö, Sweden; ^c^ Department of Sociology and Anthropology, University of Dar es Salaam, Dar es Salaam, Tanzania; ^d^ Programme in Human Sexuality, Department of Family Medicine and Community Health, University of Minnesota, Minneapolis, MN, USA; ^e^ Community Health Education Services & Advocacy (CHESA), Dar es Salaam, Tanzania

**Keywords:** Tanzania, MSM, STI, self treatment, pharmacists

## Abstract

**Background**: Previous research has shown that men who have sex with men (MSM) avoid formal healthcare services because of the fear of discrimination as homosexuality is illegal and stigmatized in Tanzania. Instead, self-treatment by medications obtained directly from pharmacies or drugstores may be common when MSM experience symptoms of suspected sexually transmitted infections (STIs) related to sexual activity with men.

**Objective**: To explore MSM’s perceptions and experiences of seeking treatment and advice from pharmacists and drugstore workers in Dar es Salaam, Tanzania, with regards to their sexual health and STI-related problems.

**Materials and Methods**: 15 in-depth interviews were conducted with MSM with experience of seeking assistance relating to their sexual health at pharmacies and drugstores in Dar es Salaam in 2016. A qualitative manifest and latent content analysis was applied to the collected data.

**Results**: Four themes related to different aspects of MSM’s perceptions and experiences of pharmacy care emerged from the analysis: (1) *Balancing threats against need for treatment* reflected informants’ struggles concerning risks and benefits of seeking assistance at pharmacies and drugstores; (2) *Identifying strategies to access required services* described ways of approaching a pharmacist when experiencing a sexual health problem; (3) *Seeing pharmacists as a first choice of care* focused on informants’ reasons for preferring contact with pharmacies/drugstores rather than formal healthcare services; and (4) *Lacking reliable services at pharmacies* indicated what challenges existed related to pharmacy care.

**Conclusions**: MSM perceived the barriers for accessing assistance for STI and sexual health problems at pharmacies and drugstores as low, thereby facilitating their access to potential treatment. However, the results further revealed that MSM at times received inadequate drugs and consequently inadequate treatment. Multi-facetted approaches are needed, both among MSM and drugstore, pharmacy, and healthcare workers, to improve knowledge of MSM sexual health, STI treatment, and risks of antibiotic resistance.

## Background

Globally, men who have sex with men suffer from discrimination in the health sector in settings with high social discrimination and where homosexuality and same-sex-behaviors are criminalized [1]. Studies from the sub-Saharan African continent have reported that MSM face prejudices, harassment and rejection by healthcare workers when they seek services [2–8]. In addition, the pervasive homophobic/homonegative climate around homosexuality has limited MSM’s access to health information and human immunodeficiency virus/sexually transmitted infections (HIV/STIs) prevention programs [9,10].

In Tanzania, same-sex sexual relations are illegal according to the penal code [11]. Previous studies have revealed that discrimination and stigma may constitute a barrier for MSM to seek care and treatment from hospitals and health centers for sexually transmitted infections (STIs) [12–15]. In a study from Dar es Salaam, Tanzania, Nyoni et al. [13] found that 14.8% of the surveyed 271 MSM reported that discrimination was a barrier to access services for HIV testing. At the same time, data indicate that HIV and STIs are concerns for this population [16,17]. Ross et al. [16] reported that 21.4% of the 200 surveyed MSM had chlamydia or gonorrhea, of which more than 90% of the infections were rectal. In another study, prevalence of self-reported STI-associated symptoms among MSM ranged from 49% with genital pain to 10% with anal sores in the sample of 200 MSM in Dar es Salaam [15]. All together, this suggests that there are potentially unmet needs for sexual health services in this population [12].

Findings from previous research in Tanzania show that obtaining drugs directly from the pharmacy or drugstore without a prescription, so-called self-treatment, might be a preferred healthcare-seeking strategy for the MSM population [12,15,18]. This may be particularly relevant for STIs, since rectal symptoms may raise suspicions that the patient is gay or has sex with other men. However, in a study using simulated client visits to pharmacies in Dar es Salaam, albeit not focusing especially on MSM, Viberg et al. [19] found that pharmacists often dispensed incorrect dosages for reported STI symptoms. Against the background of increasing trends in the incidence of antibiotic resistance – both globally and in Tanzania – inadequate drug administration is of high concern [20,21].

Several studies have focused on Tanzanian MSM’s experiences of healthcare services [12–15]. However, there is a dearth of literature on the experiences of MSM who use the services of pharmacists and drugstore workers for care and treatment of their sexual health. Based on evidence suggesting that MSM engage in self-treatment, this study sought to explore MSM’s perceptions and experiences of seeking treatment and advice from pharmacists and drugstore workers in Dar es Salaam for suspected STIs and other issues concerning their sexual health. A deeper understanding of the choices and experiences that underlie self-treatment is important in order to inform interventions that target the sexual health of MSM.

## Methods

### Study setting

The study was conducted at private pharmacies and drugstores in Dar es Salaam, the largest city in Tanzania, with a population of about four million [22]. There are two types of private pharmacies in Tanzania: Part I and Part II [23]. Part I pharmacies are licensed to sell prescription-only drugs and operated by a registered pharmacist. Part II pharmacies, or drugstores, are only authorized to sell over-the-counter medicines (i.e. drugs that do not require a prescription), and the person assisting the clients is required to have a basic medical background, for example being a nurse or pharmacy technician [23,24]. However, prescription-only drugs such as antibiotics are usually also available at unauthorized pharmacies due to problems with the enforcement of regulations [23]. Since most Part I pharmacies are available in urban areas, approximately 60–70% in Dar es Salaam only, the government has been running the accredited drug dispensing outlet (ADDO) program to build the capacity among Part II pharmacies outside urban areas to become essential drug shops [25].

### Study design

This study was approved by the Senate Research and Publication Committee at Muhimbili University of Health and Allied Sciences, Tanzania (ref. no. 2015–11/AEC/Vol.IX/102). A qualitative approach was chosen to gain access to rich and detailed descriptions of men’s perceptions and experiences of pharmacy care [26]. A semi-structured interview guide with open-ended questions was developed following a broad framework around STI history and experiences of the healthcare system and of seeking assistance from pharmacists and drugstore workers for problems related to same-sex activity. The interview guide was developed in English and translated into Swahili by a member of the research team. The Swahili version was then back-translated into English to ensure the accuracy of the translation.

### Data collection

Sixteen MSM were recruited through the assistance of a local organization working with the target group. Informants were purposively selected based on having made at least three visits to a pharmacy or drugstore for sexual health issues related to same-sex behavior and being at least 18 years old, in order to gain access to experiences of pharmacy care. The organization sent out a message about the study, including the inclusion criteria, through its e-mail directory and viva voce. Those men who were interested were invited to the premises of the organization at an agreed date for an interview. Sixteen men registered interest to participate but one was excluded due to failure to meet the inclusion criteria and the final study population was therefore 15 (see Table 1 for informants’ characteristics).

**Table 1.** Informants’ characteristics.

Informant	Age (years)	Relationship status	Living situation
**1**	24	Single	Alone
**2**	25	Single	With mother/father
**3**	25	Single	With mother/father
**4**	24	Single	Friends
**5**	27	Single	Alone
**6**	24	Single*	Alone
**7**	27	Single	Alone
**8**	28	In relationship	Alone
**9**	30	Single	With mother/father
**10**	23	Single	With mother/father
**11**	26	Single	N/A
**12**	32	Single**	Alone
**13**	21	Single	With mother/father
**14**	27	In relationship	Friends
**15**	26	In relationship	Single

*In the process of marrying a woman but informant defined himself as single.

**Previously married to a woman.

The one-on-one in-depth interviews were conducted during three weeks in 2016 in Dar es Salaam by the first or second author in English (five interviews) and when required, an interpreter was used (10 interviews). All interviews were audio-recorded, and informed consent was obtained verbally. This procedure was considered appropriate to protect the anonymity of the informants due to the sensitive political climate surrounding homosexuality. Before the interview started, informants were provided with information about the study, that they could decline answering any question and that they could discontinue the interview at any stage. No informant declined participation. A card with contact details to the responsible researcher in Tanzania was also given in case the informants had follow-up questions after the interview. During the interviews field notes were taken to capture observations and reflections, aiding the researchers in the process of understanding the data. Each interview took approximately one hour, and participation was unremunerated.

After 15 interviews there was a common understanding among the co-authors that saturation, that is, no new information continued to emerge, had been achieved in order to sufficiently cover a range of perceptions and experiences with regards to healthcare-seeking for sexual health-related issues at pharmacies and drugstores [27].

### Data analysis

All audio-recorded interviews were transcribed verbatim and translated into English by a research assistant working with the project. To process the in-depth interviews, a manifest and latent qualitative content analysis as described by Graneheim and Lundman [27] was applied. Using this approach allows both the visible (manifest content; what was said) and underlying (latent content; for example, a feeling behind the words) aspects of the text to emerge [27,28]. The analysis consisted of several steps, and throughout the analytical process each step was accompanied by a discussion among the co-authors. The first and second authors repeatedly read all transcripts to gain an in-depth understanding of the content, which in the next step was divided into units of analysis (i.e. each answer). The units of analysis were coded initially line-by-line to identify important concepts and ideas in relation to the aim. Categories were created by comparing differences and similarities across the codes and then organizing them into groups based on the unity of the code content [27]. During the last step, the categories were examined for potential themes that cut across the categories, thus representing the underlying meaning (see Table 2 for an example of the analytical process.

**Table 2.** An example of the analytical process: from meaning unit to theme.

Meaning unit	Condensed meaning unit	Codes	Category	Theme
*We are sharing information or the experiences we have of diseases with friends, giving the number and linking them to friendly pharmacists where they can go and get medical help.*	Sharing information or experiences about diseases with friends, giving number to friendly pharmacist where go for medical help	- Talking with friends about diseases - Connecting to friendly pharmacist	Using social networks of other MSM	Identifying strategies to access required services

MSM: men who have sex with men.

## Results

**Figure 1.**
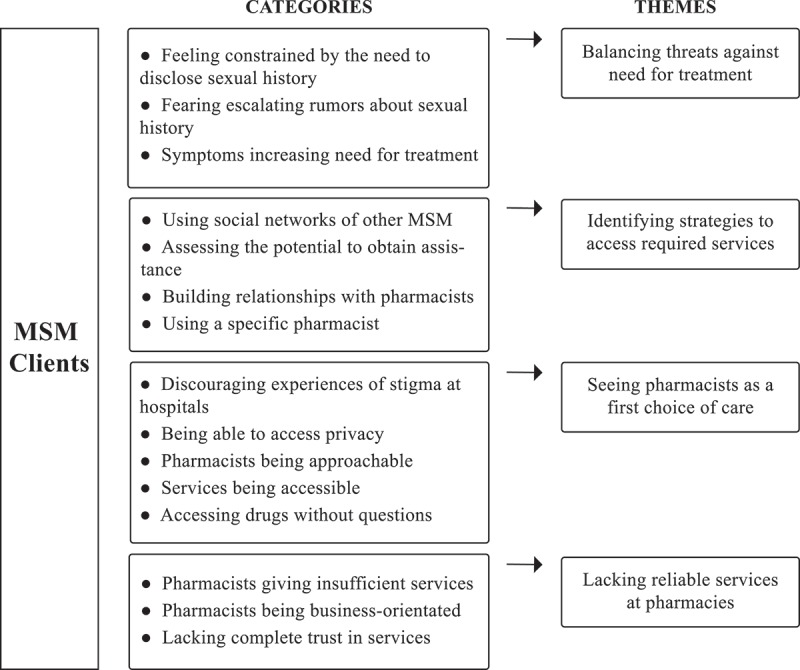
Analytical model of the results. MSM: men who have sex with men.

During the analysis of the 15 in-depth interviews four themes emerged: *Balancing threats against need for treatment, Identifying strategies to access required services, Seeing pharmacists as a first choice of care*, and *Lacking reliable services at pharmacies*. These themes illuminate the underlying meanings of the categories (Figure 1). Results are presented in the text, with themes as headings, and categories underlined within single quotation marks.

The model shows the results of the manifest and latent content analysis regarding MSM’s perceptions and experiences of using pharmacy and drugstore workers to provide sexual healthcare. Codes were aggregated into categories (manifest analytical level). Categories were examined for emerging themes (latent analytical level).

### Balancing threats against need for treatment

Coming out to a pharmacist or drugstore worker was at times necessary, as the nature of the sexual health-related problems sometimes required MSM to reveal their sexual behaviors. When reflecting on the first time they sought pharmacy care for a sexually related health problem, informants revealed being afraid of what might happen during the encounter, yet feeling driven by dire treatment needs.

‘Feeling constrained by the need to disclose sexual history’ described feelings of shame related to being MSM or gay and being afraid of pharmacists’ reactions, which informants believed could lead to the rejection of services.

There is a problem, you see, some pharmacists might react badly when they meet a gay person and not give you treatment. It’s difficult to know his reaction before you’ve met him. (Informant 14)

While MSM did not mention any discriminatory experiences by pharmacists or drugstore workers, they nevertheless had fears about revealing their sexual identity and the broader social consequences if exposed as MSM to the community. Before knowing a pharmacist or drugstore worker, informants were ‘fearing escalating rumors about sexual history’, as a potential consequence if the pharmacist or drugstore worker would gossip to others about what they had found out. An example given was ‘the story is in his hands once you tell him’ (Informant 2). At the same time, ‘symptoms increasing need for treatment’ indicated that informants reached a tipping point where the health problem left them little option other than seeking help from the pharmacist or drugstore worker.

I had an urgent problem with my genitals and there was no way out other than telling the pharmacist about my suspicions. Because he would have understood anyway that I had a sexually related problem and I really needed some treatment. (Informant 9)

Although not all STI symptoms might have necessitated the disclosure of same-sex behaviors, the findings suggest that MSM were particularly concerned about those that might be considered evidence of this.

### Identifying strategies to access required services

The fear of discrimination involved taking various precautions when selecting a pharmacist or drugstore worker. Efforts were made to identify a service provider who could be of help, and informants considered how to best approach this person in order to get the assistance required.

When reflecting upon how they accessed information about pharmacies/drugstores to approach, men described that they were ‘using social networks of other MSM’ to retrieve details about which pharmacy or drugstore to use.

We are sharing information or the experiences we have of diseases with friends, giving the number and linking them to friendly pharmacists where they can go and get medical help. (Informant 9)

However, before approaching a particular pharmacy or drugstore, informants described various strategies used when ‘assessing the potential to obtain assistance.’ This was typically achieved by visiting the pharmacy or drugstore in order to observe the venue and the service provider and by buying other items to establish contact. The strategy enabled men to make an assessment about the venue and the people who worked there, which they used to make a decision about whether they should approach the provider or not. A typical example of how men were deliberating about service potentiality was the following:

I thought that I wouldn’t be able to be free there [at the pharmacy] because many people go there to purchase drugs so you won’t find that comfort of expressing yourself. I therefore decided to go to another place. (Informant 11)

Once a pharmacist/drugstore worker had been identified, informants were ‘building relationships with pharmacists’ by engaging in small talk and returning to the pharmacy or drugstore several times.

She [the pharmacist] was astonished by my appearance first but as time went on she became used to me. I kept going there to buy some other stuff like perfumes, lotion and sometimes spending time there chatting with her. (Informant 12)

‘Using a specific pharmacist’ therefore became a crucial part of men’s healthcare-seeking strategies. Informants described that when finding a pharmacist or drugstore worker who helped them with their sexual health, they tended to come back to meet with that specific person next time they had a problem.

### Seeing pharmacists as a first choice of care

When MSM described their preference for seeking healthcare services for STI-related conditions at pharmacies and drugstores, several aspects were highlighted. Pharmacies and drugstores were the first choice of care especially for anal conditions such as abrasions and rectal pain, as these were regarded as particularly revealing of same-sex behavior. Above all, there was a perception that these service providers put less focus on men’s sexual orientation and more focus on providing services, which facilitated informants’ access to consultation and treatment.

‘Discouraging experiences of stigma at hospitals’ were offered as explanations for why informants chose pharmacists and drugstore workers as service providers for their problems.

When I go to the hospital they discriminate against me. They segregate me from the other patients and they start pointing their fingers at me. So it’s better to go to the pharmacy instead of the hospital when you’re sick. (Informant 15)

At pharmacies and drugstores, in contrast, ‘being able to access privacy’ was highlighted as an important feature. Informants described that if there were customers in the store when they arrived, they could communicate with the service provider to find out when the place was empty, enabling them access to private consultations.

Sometimes when I go and there are people in the pharmacy I tell her [the pharmacist] I will come later because I have her number. She can call me or I can call her so that I can go when the crowd is small. (Informant 9)

‘Pharmacists being approachable’ described informants’ perceptions of being listened to by the pharmacist/drugstore worker, who took time to communicate and respond to questions.

He [the pharmacist] always listens to what I have to say and if he is busy he asks me to return later so he has time for me. It helps me feel that I can share my problems and ask any question that I have. (Informant 14)

Pharmacists and drugstore workers were described as friendly, and men felt that they were valued as human beings. Furthermore, ‘services being accessible’ was repeatedly stated. Informants discussed the proximity to pharmacies and drugstores and their longer opening hours compared with hospitals. Thus, time-saving and accessibility were key features that men emphasized regarding their preferences for pharmacy care.

MSM are familiar with the pharmacies because they can be reached easily and they can go even during the night because the hospitals that we normally go to are closed by 3 am. (Informant 10)

‘Accessing drugs without questions’ was another decisive aspect when using pharmacies. The option of being able to remain anonymous to obtain drugs without a prescription was emphasized – in particular when using pharmacists and drugstore workers with whom informants were unfamiliar.

I was given the medicine without any question. He [the pharmacist] didn’t want to know who I was or whatever. (Informant 3)

### Lacking reliable services at pharmacies

Despite the fact that pharmacists and drugstore workers were used as care providers in situations when men could not seek services at hospitals due to fears of being victimized, men also described barriers that interfered with their ability to obtain quality care. These were mainly related to profit-making aspects of pharmacy services but also to a general lack of STI-specific knowledge, which lessened their trust in these services.

‘Pharmacists giving insufficient services’ referred to the perception that pharmacists and drugstore workers lacked comprehensive knowledge about STI conditions and consequently failed to give accurate treatment. This resulted in incorrect treatment and informants did not recover fully.

I told the pharmacist that things are not right in my genital parts: ‘I think I have fungus.’ He checked me and recommended drugs that I should take and I had some injection. Out of 100% I can say that the service was satisfactory by 65%. I was not very comfortable since the treatment was not sufficient. (Informant 11)

Informants stressed the fact that pharmacists and drugstore workers relied on an income and ‘pharmacists being business-oriented’ emerged as a category. Informants reported that they had been offered the opportunity to buy half-dosages when lacking sufficient resources to buy a full dosage.

You can tell the pharmacist if you can’t afford to pay for all of them [the drugs] and they choose for you what you can afford. (Informant 15)

Men also felt that pharmacists and drugstore workers tried to sell them additional drugs, which were believed to be unnecessary given the STI symptoms they suffered from. Informants attributed this to the fact that these service providers wanted to make money, and this contributed to MSM ‘lacking complete trust in services’ provided by pharmacists and drugstore workers.

## Discussions

The current study is one of the first to explore Tanzanian MSM’s perceptions and experiences of seeking help at pharmacies and drugstores for issues concerning their sexual health, including suspected STI symptoms. Men described perceptions and experiences of pharmacy care that were both complex and contradictory; a perception that the service provider tried to accommodate their needs was accompanied by insecurity about administered drug dosage and treatment. This is of great concern both in terms of the right to adequate and timely healthcare assistance but also in terms of the consequences of incorrect drug management. Improper treatment of STI infections runs the risk of contributing to the development of antimicrobial drug resistance. Given that a significant number of MSM in Tanzania also have parallel sexual relations with women, this is a problem that concerns the wider population [29].

Barriers for using formal healthcare due to stigma and discrimination were reported, and men felt limited in their options concerning where to seek assistance. Similar barriers have also been found in other studies from Dar es Salaam [12,13,15]. In a study of 200 MSM in Dar es Salaam, 71–90% reported experiencing ‘not polite’ attitudes from healthcare workers when informing about STI symptoms [15]. Our results that pharmacists and drugstore workers were perceived as being accessible and as providing services for MSM’s sexual health-related problems are corroborated by other studies in Tanzania [12,18]. Larsson et al. [18] in a study of pharmacists in Dar es Salaam, found that the bonds created between the pharmacists and their MSM clients enabled service provision despite personal reservations against same-sex practices. Men in our study referred to the fact that they knew their pharmacist, which aided them in the process of seeking treatment for their sexual health. The open nature of a pharmacy enables clients to observe and form an opinion over time without exposure, and to ‘choose’ a pharmacist who appears accepting (in contrast to hospital clinics where choice of provider is not possible). The social context in which the consultation takes place has previously been explored in other studies in sub-Saharan Africa [30,31]. The decreased social distance between the client and the pharmacist, compared with that between a physician and a patient, appears to be a motivating factor behind the preference for self-treatment – in particular concerning STIs, which are associated with shame and other negative feelings [32]. This might also be relevant for our findings and could possibly explain why MSM were able to develop personal relations with pharmacists. Furthermore, the development of these relations appeared to be facilitated by the accessibility of the pharmacies and drugstores, mainly their longer opening hours and geographical location, allowing men to ‘drop in’ a number of times and eventually to establish a relationship with a specific pharmacist. However, there has been a recent rise in the Tanzanian government’s anti-gay rhetoric, and in 2016 it temporarily suspended MSM outreach programs [33,34]. If the climate around homosexuality continues to develop adversely this may also have implications for other services targeting the MSM population, including pharmacy care, as providers might fear repercussions from assisting MSM clients.

Nevertheless, informants also described instances when they had been given ineffectual medication or incorrect dosages. One interpretation is that pharmacists wanted to make a sale, and if lacking the drug needed, or wanting to sell something more expensive, they recommended other drugs in order to make a profit. Alternatively, they lacked proper knowledge about STI management. The current density of pharmaceutical personnel in Tanzania is low; in 2012 there were only 0.013 trained pharmacists per 1000 population [35]. This means that many clients, including MSM, rarely come in contact with a trained pharmacist, and instead seek assistance from clerks or drugstore workers who lack pharmaceutical training. In order to counteract the leniency concerning adherence to the existing drug regulations, training of drugstore personnel who lack adequate pharmaceutical education is of importance. Untreated STIs could not only result in detrimental health outcomes but also increase the risk for contracting HIV or contribute to the spread of antimicrobial drug resistance. Initiatives such as the government-accredited drug dispensing outlets program (the ADDO program) where drugstore staff receive basic pharmaceutical training and are sensitized to refer complicated cases to health facilities, should ideally also be expanded to urban areas to cover the many Part II pharmacies, for example drugstores [25,36]. However, to enable an adequate response, it is equally important to ensure that healthcare workers are enabled to respond to the sexual health needs of MSM patients. Furthermore, in light of our findings that confidentiality, privacy and trust were highly prioritized by informants, as also confirmed by other studies targeting African MSM [3,37], these aspects should be emphasized in the training of healthcare workers.

MSM frequently referred to their social networks as important sources of healthcare information. In a previous investigation of the social networks of MSM in Tanzania, Ross et al. [38] found that the mean size of personal networks in Dar es Salaam was 12. This study offers further insights into these networks, which enabled informants to get access to information about what drugs to obtain when suffering from certain STI symptoms. In the absence of political support and enabling policies towards establishing gay-friendly services, the social networks of MSM could be used to deliver sexual health education messages, including the importance of a proper diagnosis of STI symptoms before treatment. Network leaders could, for example, be trained in prevention and treatment information and guided to convey the information to members of their networks [39].

### Methodological considerations

This study contributes new knowledge about Tanzanian MSM’s experiences of pharmacy care related to their sexual health. However, the current study was localized to Dar es Salaam, and therefore might be limited in scope. The perceptions and experiences of the informants are unique to the context in which the data were collected, and the findings cannot be generalized to other MSM populations. However, given the strong stigma of homosexuality in the region, it is probable that our findings are transferable to other, similar, settings where stigma is prevalent [40]. Several measures were taken to ensure the study’s trustworthiness. The study focused on the perspectives of MSM and by engaging with informants and reflecting on the findings, an enhanced understanding of these perspectives was obtained [41]. To deepen our understanding of the data, representatives from the gay community in Dar es Salaam were consulted regarding the cultural context and local expressions, which further contributed to the credibility of the findings [40]. During the interviews it was always made clear to informants that no right answer existed for the questions asked, and informants were encouraged to speak their minds. Debriefing sessions with the research team were held regularly during each step of the analysis (codes, categories and themes) to discuss and seek agreement on the patterns and themes that emerged [40]. The reflective field notes were instrumental in clarifying the researchers’ own perceptions and ideas, thereby minimizing potential bias during data analysis and enhancing confirmability. The use of quotations to illustrate the categories lends transparency and enables the reader to understand the interpretation of the findings. Furthermore, the detailed account of the study process allows future researchers to repeat the work (i.e. dependability).

A limitation of the study is that all contact persons were referred to by the informants as ‘pharmacists’, and thus, it is unknown whether the men’s experiences concerned contact with a pharmacist or with a drugstore worker, more specifically. Furthermore, although the sample represents a range of perceptions and experiences of pharmacy care, it consisted of relatively young men (between 21 and 32 years old), the majority of whom were not in a relationship. Older MSM, or MSM in relationships, may have different perceptions and experiences of pharmacy care. It may also be noted that the study was conducted in Dar es Salaam, which is the largest city in Tanzania, and the situation could be different in the rural areas where the density of pharmacies and drug stores is lower [42].

Since Swahili is a foreign language to the research team, we used local interpreters for 10 of the interviews. This was a deliberate choice to secure that the informants could fully understand our questions and develop their answers, which also helped achieve trustworthy answers from the informants [40]. Yet, using interpreters could have influenced the outcome of the interviews since the information might have been altered during the process of translation. To mitigate this risk, we engaged professional translators, who were provided with the original interview recordings in Swahili. Furthermore, the inclusion prerequisite (i.e. a minimum of three previous visits to a pharmacy or drugstore for sexual health issues related to same-sex behavior) might have influenced the perceptions that were reported. Finally, there is a possibility that the gender of the interviewers (women) could have influenced the interview situation and that informants might have felt more comfortable discussing the issues raised with someone of the same gender. However, to address such risks, the interviewers explained their background and familiarity with the topic to each informant, and emphasized the confidentiality of the study.

## Conclusions

The study describes the perceptions and experiences of Tanzanian MSM when seeking sexual healthcare at pharmacies and drugstores. Despite the localized nature of the study, it offers insights into the situation of a vulnerable population and could be used as a starting point for targeted interventions towards MSM and pharmacists/drugstore workers. Multi-facetted interventions are required that address healthcare workers as well as pharmacists and drugstore workers. Clearly, the MSM population itself has an important role to play and needs to be actively involved in the design, execution and evaluation of interventions that concern their sexual health.

## Paper context

The stigma of homosexuality leads many MSM to resort to self-treatment when suspecting STIs. Incorrect management of STIs could have detrimental health effects and result in antibiotic resistance. Our research provides MSM’s perceptions and experiences of seeking treatment at pharmacies and drugstores for sexual health-related problems. Findings from this study could be a starting point for interventions targeting MSM, pharmacists and healthcare workers in Tanzania.

## Thematic interview guide – English

1. Would you like to tell me about your experiences of seeking care at a pharmacy when having sexual health-related questions or suffering from a sexual health related illness?

2. What would you say guides your decision to seek care at the pharmacy rather than the health care system when you are sick or not feeling well?

Probe

*Are there certain things that are extra important to you when you make a choice of where to go when you are sick?*

3. Could you describe the first time you visited a pharmacy or drugstore for service provision regarding your sexual health – what made you take that decision?

Probes

*Was there a particular incident that affected you?*

*Did your friends talk about pharmacists and drugstore workers as sources of assistance?*

4. I would like to know a little bit more about this first visit; could you tell me what you did to get the service you needed?

Probes

*How was the service?*

*How did you feel?*

5. According to you, what are the main differences and similarities between how pharmacists and healthcare workers treat you as an MSM?

Probe

*Why do you think this is so?*

6. Could you describe your relationship with the pharmacist that you often go to?

Probes

*Are you close? In what way?*

*How does your relationship with the pharmacist affect your choice of care?*

*What made you choose him or her as a main provider?*

7. Have you disclosed your sexual orientation to the pharmacist that you often go to?

Probes

*If yes, describe to me when and how you did it?*

*What made you disclose it?*

*What was the reaction?*

*If no, why haven’t you disclosed it?*

8. Could you tell me about what the environment is like at the pharmacy or drugstore where you usually go?

Probes

*Staff?*

*Other clients?*

*Size of the pharmacy?*

9. If you come to the pharmacy and there are a lot of people, what do you do?

Probes

*Do you stay and wait for your turn or do you leave?*

*What does the pharmacist do if he/she sees that you come and it’s crowded?*

## Healthcare services

In Tanzania, as well as in other countries, sexually transmitted infections are a widespread problem. I would like to talk more about this issue and also remind you that everything you say to me is completely confidential and will not be linked back to you. It is important knowledge for us in order to inform future interventions in order to improve the sexual health among MSM.

10. When you need information about sexual health, such as safe sex, HIV, sexually transmitted infections, and so on, where do you seek this information?

Probes

*Why would you look there?*

*Any specific reasons?*

*In what ways does this information relate to your own health needs?*

11. Could you describe to me what are the most important things for you when you seek services related to your sexual health?

Probes

*Confidentiality and privacy?*

*That the healthcare provider is knowledgeable?*

*The environment?*

12. What are the unique healthcare needs that you as a man who prefers to have sex with other men has when it comes to STI?

13. When you suspect that you have an STI or any other kind of sexual health problem, what do you do?

Probes

*What roles do your friends play?*

*What role does the Internet play?*

14. What are the main challenges that you see when seeking assistance for STI-related problems at a pharmacy or drugstore?

Probes

*Are there certain challenges that you face that a man who is only together with women may not have? If yes, what are these?*

15. How old are you?

16. Do you currently work or study? If yes, what do you study/where do you work?

17. What is your living situation? Alone, with family, friends?

18. How is your personal life? Married, committed relationship, single?

## Thematic interview guide – Swahili

1. Ningependa kujua uzoefu wako wa kufuatilia huduma ya afya pale unapokuwa na maswali yanayohusiana na matatizo ya kiafya ya zinaa au pale unapokuwa unasumbuliwa na ugonjwa wa zinaa?

2. Ni nini utasema kinachangia maamuzi yako ya kupata huduma katika phamasi na sio kwa mtoa huduma ya afya pale unapokuwa mgonjwa au hujisikii vizuri?

Dodosa

*Je kuna vitu ambavyo ni muhumu sana kwako unapofanya maamuzi wapi unataka kwenda unapokuwa unaumwa?*

3. Unaweza kunielezea mara ya kwanza ulipokwenda katika phamasi au duka la dawa kwa hudumu kuhusiana na magonjwa ya zinaa, nini kilikufanya kuchukua uamuzi huo?

Dodosa

*Je kulikuwa na tukio lolote lililokuadhiri?*

*Je rafiki zako huwa wanawazungumzia maphamasia au mahudumu wa maduka ya dawa kama sehemu ya kuweza kupata msaada?*

4. Ningependa kujua zaidi kuhusu tembeleo lako la kwanza, unaweza kuniambia ulifanya nini ili kupata hudumu uliyotaka?

Dodosa

*Huduma zilikuwaje?*

*Ulijisikiaje?*

5. Kwa mtazamo wako kunatofauti au kunafanana vipi kati ya jinsi mphamasia na mhudumu wa afya wanavyowachukulia wanaume wanaofanya mapenzi na wanaume?

Dodosa

*Kwanini unadhani ni hivyo?*

6. Je unaweza kuniambia kuhusu uhusiano wako na mphamasia uneyeenda kwake mara kwa mara?

Dodosa

*Je mna uhusiano wa karibu? Kwa namna gani?*

*Je uhusiano wako na mphamasia unaathiri vipi maamuzi yako juu ya hudumu unayotaka?*

*Nini kilikufanya umchague yeye?*

7. Je umeshamwambia mphamasia unayeenda kwenye duka lake mara kwa mara kuhusu tabia yako ya kigono?

Dodosa

*Kama ndio nieleze lini na ulifanyaje?*

*Nini kilikufanya uweze kumwambia*

*Je alilichukuliaje?*

*Kama hapana, kwanini hujaweka wazi?*

8. Je unaweza kuniambia kuhusu mazingira yalivyo katika pharmasi au duka la dawa?

Dodosa

*Wahudumu*

*Wateja wengine*

*Ukubwa wa phamasi*

9. Je ukifika katika duka la dawa na ukakuta watu wengi, huwa unafanya nini?

Dodosa

*Je huwa unakaa na kusuburi zamu yako au huwa unaondoka?*

*Je mtoa huduma/mfamasia huwa anafanya nini pale anapoona umekuja lakini kuna wateja wengi?*

## Healthcare services

Tanzania kama ilivyo katika nchi nyingine magonjwa ya zinaa yameenea sana. Ningependa kuzungumzia zaidia suala hili na kukumbusha pia kuwa yote utakayo kuwa unaniambia yatabaki kuwa siri na hayatahushishwa na wewe. Taarifa hii ni muhimu kwetu ili tuweze kupanga mikakati ya kuboresha afya ya uzazi kwa wanaume wanaofanya mapenzi na wanaume.

10. Unapokuwa unahitaji taarifa juu ya afya ya uzazi, kama vile ngono salama, Ukimwi, magonjwa ya ngono, ni wapi huwa unatafuta taarifa hiyo?

Dodosa

*Kwanini unatafuta huko?*

*Kuna sababu maalumu?*

*Je taarifa hiyo inahusiana vipi na mahitaji yako ya kiafya?*

11. Unaweza kunielezea ni vitu gani vya muhmu unapozingatia pale unapokuwa unafuatilia/kutafuta matibabu yanayohusiana na afya yako ya uzazi?

Dodosa

*Usiri?*

*Mhudumu wa afya anaufahamu?*

*Gharama?*

*Upatikanaji wa madawa*

*Mazingira?*

12. Je kuna mahitaji gani ya kiafya ambayo wewe kama mwanamume anayefanya mapenzi na wanaume unayaona kuhusiana na magonjwa ya zinaa?

13. Unapohisi unaugonjwa wa zinaa au ugonjwa wowote unaohusiana na ngono huwa unafanya nini?

Dodosa

*Rafiki zako wanajukumu gani?*

*Mtandao wa inteneti una nafasi/jukumu gani?*

14. Je unakutana na changamoto gani wakati unatafuta huduma katika phamasi au maduka ya dawa kuhusiana na maradhi/magonjwa ya zinaa?

Dodosa

*Je kuna changamoto zozote wewe unakabiliana nazo ambazo mwanamume mwenye mahusiano na wanawake hakutani nazo? Kama ndiyo, changamoto hizo ni zipi?*

15. Una miaka mingapi?

16. Kwa sasa unasoma au unafanya kazi? Kama ndiyo una soma wapi/unafanya kazi wapi?

17. Unaishi na nani? Mwenyewe, na familia au marafiki?

18. Je maisha yako ya mahusiano yakoje? Umeoa, uko katika mahusiano au bado uko pekee yako?
